# Erenumab in highly therapy-refractory migraine patients: First German real-world evidence

**DOI:** 10.1186/s10194-020-01151-0

**Published:** 2020-07-03

**Authors:** Armin Scheffler, Olga Messel, Sebastian Wurthmann, Michael Nsaka, Christoph Kleinschnitz, Martin Glas, Steffen Naegel, Dagny Holle

**Affiliations:** 1 Department of Neurology, West German Headache Center, University Hospital Essen, University Duisburg-Essen, Hufelandstr. 55, 45147 Essen, Germany; 2 Department of Neurology, Division of Clinical Neurooncology, University Hospital Essen, University Duisburg-Essen, Hufelandstr. 55, 45147 Essen, Germany; 3 Department of Neurology, Martin Luther University Halle-Wittenberg, University Hospital Halle, Ernst-Grube-Str. 40, 06097 Halle (Saale), Germany

**Keywords:** 1. migraine, 2. erenumab, 3. therapy, 4. real-world, 5. CGRP antibody

## Abstract

**Background:**

Calcitonin gene related peptide (CGRP) monoclonal antibodies (mAB) are the first specific migraine prophylactic medication. Erenumab is the only CGRP mAB targeting the CGRP receptor. Clinical data regarding efficacy and tolerability of erenumab in highly therapy-refractory patients are not available, yet, although many patients treated with CGRP mAB under real world conditions can be considered as highly therapy-refractory.

**Methods:**

Clinical routine data of highly therapy-refractory migraine patients treated with erenumab 70 mg for 3 months between November 2018 and December 2019 in the West German Headache Center, University Hospital Essen, Germany, were analysed. Monthly migraine days (MMD), monthly headache days (MHD) and days of acute medication intake (AMD) were assessed. Statistical analysis was performed using the Wilcoxon test. Descriptive statistics were performed to evaluate changes of vegetative symptoms, acute medication response, side effects, as well as treatment satisfaction.

**Results:**

Complete clinical data were available for 26 episodic (EM) and 74 chronic (CM) migraineurs. Sixty-six % (*n* = 49) of CM patients had an additional medication overuse headache (MOH). After 3 months 57.7% of EM patients and 41.9% of CM patients had a 50% or greater reduction of MMD. The mean number of MMD was reduced by 3.43 (SE 1.26) in EM, and by 4.72 (SE 0.87) in CM. Thirty-nine patients (52.7%) returned from chronic to episodic course of migraine. After 3 months, 23 patients (46.9%) were not suffering from a MOH anymore.

**Conclusions:**

Erenumab seems to be a promising therapeutic option in highly therapy-refractory migraine patients.

**Trial registration:**

Retrospective registered.

## Background

Migraine is the 3rd most prevalent illness in the world and one of the main causes of disability [1]. So far available non-specific prophylactic drug medication was often poorly tolerated and not effective in every patient. Recently, monoclonal CGRP (calcitonin gene related peptide) antibodies (mAB) have become the first specific migraine prophylaxis, which has shown its efficacy in large phase III studies in the treatment of episodic (EM) and chronic migraine (CM) with or without medication overuse headache (MOH) [2–4] and is now available worldwide for migraine treatment.

In Europe, three CGRP mAB, erenumab (Aimovig®, Novartis, Basel, Switzerland), fremanezumab (Ajovy®, Teva, Petach Tikva, Israel) and galcanezumab (Emgality®, Lilly, Indianapolis, USA) are approved for migraine prevention in patients suffering from at least four migraine days per month and recommended for migraine prevention by current European guidelines [5]. Erenumab is the only substance targeting the CGRP receptor instead of the molecule itself. Thus far, no clear differences in efficacy and tolerability between the individual mABs could be identified.

Clinical trials were only performed in migraine patients who had failed up to four prophylactic medications in the past [2–4]. Although there are indications of good effectiveness under real-world conditions [6, 7], data on highly therapy-refractory patients that had failed more than four prophylactic treatments are missing, yet.

We present now clinical data on this highly therapy-refractory migraine population treated with erenumab under real world conditions.

## Methods

We retrospectively analysed routine clinical data of EM and CM patients presented at the West German Headache Center, Department of Neurology, University Hospital Essen, Germany between November 2018 and December 2019. The analysis was approved by the independent ethics committee of the University Hospital Essen. Patients meeting the following criteria were included in the analysis: a) EM/CM patients with at least 4 migraine days a month according to the current diagnostic criteria of the International headache classification (ICHD-3 [8]), b) documented history of the last 90 days prior starting erenumab therapy regarding monthly migraine days (MMD), monthly headache days (MHD) and monthly intake of acute medication (AMD), c) completion of a 90 days treatment interval with monthly 70 mg erenumab d) available complete clinical data including headache diaries and side effects. A paper-based headache diary was used. MMD, MHD and AMD are the average monthly mean values over the respective total observation period of 90 days. A headache day was defined as a day with any kind of headache, a migraine day was defined by patients when they had severe pain, migraine pain characteristics (pulsating, one-sided pain), aura symptoms, vegetative symptoms like phono- or photophobia, nausea, vomiting, need for rest, or when triptans were taken. Most patients answered questionnaires regarding different aspects of migraine: intensity of migraine (*n* = 95), duration of the migraine attack (*n* = 90), effect of acute therapy (*n =* 90), effect on the aura (*n =* 90), need for rest (*n =* 93), dizziness (*n* = 92), nausea (*n =* 93), phono- and photophobia (*n =* 93) as well as therapy satisfaction (*n =* 93). Due to reasons of reimbursement by the German statutory health insurance, all treated patients had tried at least 5 (when EM) and 6 (when CM) approved prophylactic drugs previously without sufficient treatment effects, had discontinued those due to side effects, or were not eligible for intake due to contraindication. Approved drug classes were the following: betablockers (metoprolol or propranolol), tricyclic antidepressants (amitriptyline), calcium channel blockers (flunarizine), anticonvulsants (topiramate and valproic acid) and for CM additionally onabotulinumtoxin A. The pre-existing medication taken for other indications or migraine prophylaxis were not changed and on a stable dosage at least 6 weeks prior to treatment start. No other medication with a potential disease modifying effect was started during the observational period. Data were analysed using SPSS software (IBM SPSS Statistics for Windows, Version 25.0. Armonk, NY: IBM Corp). Wilcoxon’s test was used to compare MMD, MHD, and AMD before and after treatment. The test procedure was two-sided, Bonferoni’s method for multiple comparisons was set at *p* <  0.05/3 = 0.016. Patient reported outcomes were analysed descriptively.

## Results

Clinical data of a total of 100 migraine patients were analysed. Details regarding demographic data and numbers of MMD, MHD, and AMD before and after treatment are stated in Table 1. In brief, most of the patients were female (82%; *n =* 82), 26% (*n* = 26) suffered from EM, 74% (*n* = 74) from CM. Sixty-six % (*n* = 49) of CM patients had an additional MOH.

**Table 1:** Patients` characteristics and treatment response

**A) Episodic migraine (*n*=26)**					
Age, y	52.9 (SD 9.1)		Sex, female : male		23 : 3
	**Before treatment, d**	**After 3 month, d**	***P*-value**	**Change from baseline, d**	**50% response, %**
MMD (SD)	9.42 (2.91)	5.99 (5.43)	< 0.001	-3.43 (SE 1.26)	57.7
MHD (SD)	10.40 (2.47)	7.06 (5.46)	0.001	-3.33 (SE 1.17)	46.2
AMD (SD)	10.22 (4.69)	5.74 (5.07)	<0.001	-4.48 (SE 1.27)	61.5
**B) Chronic migraine (*n*=74)**					
Age, y	45.8 (SD 12.9)		Sex, female : male		59 : 15
MOH (before treatment), n	49		MOH (after 3 month), n		26
	**Before treatment, d**	**After 3 month, d**	***P*-value**	**Change from baseline, d**	**50% response, %**
MMD (SD)	15.69 (6.64)	10.97 (7.81)	<0.001	-4.72 (SE 0.87)	41.9
MHD (SD)	21.27 (5.5)	15.8 (8.9)	<0.001	-5.47 (SE 0.86)	32.4
AMD (SD)	11.62 (6.14)	8.86 (5.97)	<0.001	-2.76 (SE 0.83)	27.0

Data before treatment reflect the 3 months before start of erenumab treatment. (*MMD* monthly migraine days, *MHD* monthly headache days, *AMD* monthly days of intake of acute migraine medication, *SD* standard deviation, *SE* standard error, *y* years, *d* days)

**Fig. 1:**
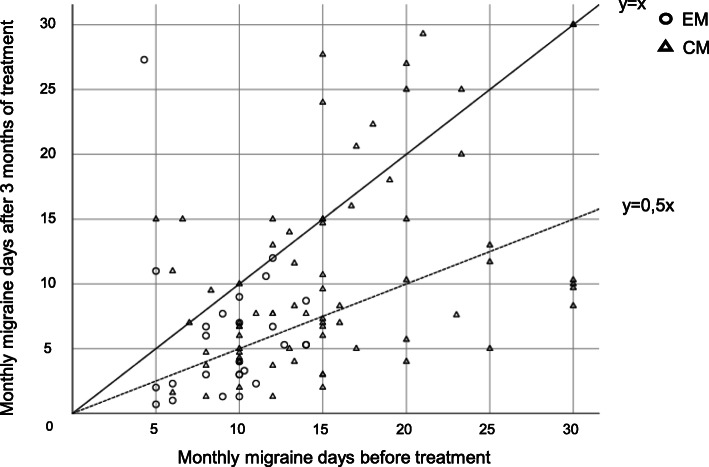
Therapy response after 3 months of erenumab treatment. Number of monthly migraine days before and after three months of treatment with erenumab. Every plotting symbol represents a patient with EM (circle) or CM (triangle). All symbols on the function y = 0.5x and below show a reduction of at least 50%. The symbols below the function y = x represent patients who still have less monthly migraine days after 3 months of treatment, all symbols on the function or above can be considered as non-responder

After 3 months of erenumab therapy 57.7% of EM patients (*n* = 15) and 41.9% of CM patients (*n* = 31) had at least a 50% reduction in MMD (Fig. 1). Fifty-three % of CM patients (*n* = 39) returned from chronic to episodic course of migraine. After 3 months, 46.9% of MOH patients (*n* = 23) were not suffering from MOH anymore.

**Fig. 2:**
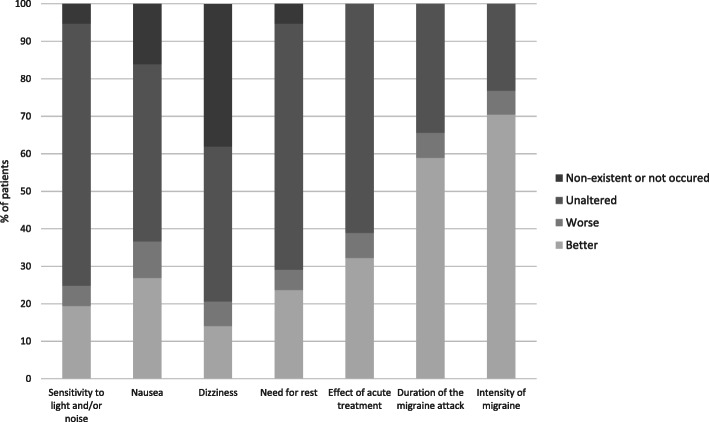
Distinct treatment responses regarding migraine characteristics. Patient reported subjective treatment response after three months of erenumab therapy. Migraine-associated vegetative symptoms, dizziness, need for rest and effects of acute medications stayed unaffected in the majority of patient, while duration and intensity of migraine improved

Subjective improvement of migraine intensity and duration was reported by considerably more than half of the patients (*n* = 67, 70.5% and *n* = 53, 58.9%, respectively). Subjective treatment effects on migraine associated vegetative symptoms were rather small. Efficacy of acute medication was largely unchanged under therapy (Fig. 2).

Self-reported general satisfaction with therapy was high (very satisfactory/satisfactory 61,3% (*n* = 57); moderately satisfactory 20,4% (*n* = 19); unsatisfactory/very unsatisfactory 18,3% (*n* = 17)).

Out of 100 patients 42% (*n* = 42) reported side effects. These 42 patients mainly complained digestive problems or constipation (23,8%, *n* = 10), injection side skin symptoms or itching (23,8%; *n =* 10), fatigue or a feeling of exhaustion (16,7%, *n =* 7), and insomnia (9,5%, *n =* 4). One patient discontinued treatment due to severe constipation.

## Discussion

Our clinical data show efficacy and tolerability of CGRP mAB under real world conditions in a German tertiary headache center. Data on this highly therapy-refractory patient population were not available, yet. The results of our analysis look very promising, especially considering the fact that no other approved drug therapies have been available for this patient population.

Efficacy of erenumab in highly therapy-refractory patients is comparable to results from clinical trials of patients that had failed fewer prophylactic medications prior treatment. Under trial conditions migraine days went down between 1.8 and 3.7 days/month [2, 9, 10] compared to a reduction of 3.43 days/month in EM under real world conditions; MMD 50% responder rates were between 30% and 43.3% [2, 9, 10] compared to 57.7% in this real-world analysis. Results from the phase II erenumab CM trial reported a reduction of 6.6 days/month and a 50% responder rate of 40% [11] which is comparable to our real world data showing a reduction of 4.72 days and a 50% responder rate of 41.9%. The slightly better treatment effect under real-world conditions compared to previous clinical study results may be explained by extraordinarily high patient expectations regarding the effectiveness of the drug and the lack of a placebo arm. Further data will show to which extent this effect stays stable during long-term treatment.

Compared to the clinical studies, similarly high rates of side effects were found and no serious side effects occurred [2, 9, 12] supporting a good tolerability of erenumab.

Many patients with highly therapy-refractory migraine suffer from additional MOH, in our real-world population 49% of all analysed patients. Our data show that erenumab could also be effective in patients with additional MOH although medication overuse was not stopped prior therapy. Our observations confirm the data of the subgroup analysis of the CM Phase II erenumab study [12], also showing the efficacy of erenumab in MOH. Nevertheless, a migraine day was defined inter alia when migraine specific acute drug intake (triptane) was needed (as described in the method part). Our data can only show a reduction of acute drug intake as well as an improvement of MOH at the same time. We are not able to distinguish, if erenumab has a direct effect on MOH or if the reduction of AMD leads to an improvement of the MOH.

Besides reduction of frequency and acute medication intake, the greatest therapeutic effect was seen in reduction of intensity and duration of migraine attacks. The therapeutic effect of erenumab seems to be less pronounced regarding vegetative accompanying symptoms of migraine. For the majority of our patients, migraine-associated phonophobia, photophobia and nausea remained unchanged. It can only be assumed that vegetative accompanying symptoms are mainly driven by central mechanisms and therefore less influenced by the peripheral acting CGRP antibody whereas migraine pain intensity and frequency might be more peripherally modulated [13].

Shortcoming of this single-center experience are mainly its retrospective nature and that data are mainly based on purely subjective reports of patients. Nevertheless, these data are important from our point of view, as better evaluations regarding headache of this predominantly treated patient collective are still missing. Additionally, patients in our patient population received the lower erenumab dosage of 70 mg instead of the now available 140 mg dosage. There is some evidence that difficult-to-treat patients who failed prophylactic medication in the past may benefit more from 140 mg compared to 70 mg erenumab [14]. However, there are no data of direct comparison between 70 and 140 mg dosage. Whether patients could benefit more from a higher dosage cannot be answered at present.

## Conclusion

Our data show that erenumab is a promising therapy option in highly therapy-refractory migraine patients with or without MOH. Real-world efficacy and tolerability of erenumab seems to be comparable with results from clinical trials, even in more therapy-refractory patients than those treated in clinical studies. Future applications will show whether our results can be confirmed for larger numbers of patients and for CGRP mAB in general.

## Data Availability

The datasets used and/or analysed during the current study are available from the corresponding author on reasonable request.

## Abbreviations

AMD: Monthly intake of acute medication
CGRP: Calcitonin gene related peptide
CM: Chronic migraine
EM: Episodic migraine
ICHD-3: International headache classification
mAB: Monoclonal antibody
MHD: Monthly headache days
MMD: Monthly migraine days
MOH: Medication overuse headache
SE: Standard error
